# Probe-caught spontaneous and deliberate mind wandering in relation to self-reported inattentive, hyperactive and impulsive traits in adults

**DOI:** 10.1038/s41598-018-22390-x

**Published:** 2018-03-07

**Authors:** Gizem Arabacı, Benjamin A. Parris

**Affiliations:** Faculty of Science and Technology, Department of Psychology, Bournemouth University, Poole, UK

## Abstract

Research has revealed a positive relationship between types of mind wandering and ADHD at clinical and subclinical levels. However, this work did not consider the relationship between mind wandering and the core symptoms of ADHD: inattention, hyperactivity and impulsivity. Given that the DMS-V attributes mind wandering to inattention only, and that only inattention is thought to result from impairment to the executive function linked to mind wandering, the present research sought to examine this relationship in 80 undiagnosed adults. Using both standard and easy versions of the Sustained Attention to Response Task (SART) we measured both spontaneous and deliberate mind wandering. We found that spontaneous mind wandering was related to self-reported inattentive traits when the task was cognitively more challenging (standard SART). However, hyperactive and impulsive traits were related to spontaneous mind wandering independent of task difficulty. The results suggest inattentive traits are not uniquely related to mind wandering; indeed, adults with hyperactive/impulsive traits were more likely to experience mind wandering, suggesting that mind wandering might not be useful diagnostic criteria for inattention.

## Introduction

Mind wandering has been defined as a shift of attentional resources from an external task toward internal thoughts, thus competing with the cognitive demands of the primary task for limited resources^1,2^. However, an emerging body of literature suggests conflicting hypotheses as to the nature of mind wandering. Under another view, task-unrelated thoughts (TUTs) are not resource demanding and are automatically and continually generated. Under this view, mind wandering occurs because executive control mechanisms fail to inhibit task unrelated thoughts representing a failure in executive control^3–5^.

The evidence for the executive failure view^3–5^ comes from the finding that those with high working memory capacity exhibit less mind wandering than those with low working memory capacity when the task is cognitively challenging enough^4,6,7^. Individuals with high working memory capacity are thought to be more able to adjust their levels of mind wandering when a task is challenging so that mind wandering would not hinder task performance. Individuals with low working memory capacity in contrast fail to adequately combat interfering thoughts and as a result, when attentional focus is needed for the primary task, their thoughts stay on-task less. Those with high working memory capacity readily inhibit such thoughts to achieve task performance. For example, during the Sustained Attention to Response Task (SART)^8^, a commonly used task to sample mind wandering events, task difficulty has been shown to interact with the level of mind wandering^9^. The SART presents participants with randomly presented digits (from 1 to 9) that participants must closely monitor and press a key on every trial except for when the number 3 appears. A view explaining the demands of the SART, the underload view, suggests that the struggle in performing SART is driven by the monotonous nature of the task^10^. Following this view, the SART is not a cognitively effortful which leads to frequent occurrences of attentional lapses^10^, making it difficult to stay on task^8^.

An alternative view, the cognitive overload view of SART^11^, suggests that the task requires constant monitoring of stimuli over a relatively extended period. Frequent attentional lapses resulting from the monotonous nature of the task creates cognitive overload and thus problems inhibiting the responses to the target stimuli. Consistent with the overload view, increased errors were observed with higher task demands during SART^12–16^. Indeed, Seli, Risko and Smilek^9^ employed both the standard SART in which the digits appear randomly and an easier version of the SART in which the digits appear sequentially as a way to manipulate task difficulty. Since the order of the digits was predictable, it is reasonable to argue that the sequential SART would be easier to monitor the target digit.

The literature has also stressed the heterogeneous nature of mind wandering^9^. Although the literature has generally assumed that mind wandering means spontaneously generated thoughts, participants, when probed, report intentional engagement of mind wandering^17^. This *deliberate* mind wandering is an effortful, intentional engagement of unguided thoughts, whereas spontaneous mind wandering refers to experiencing unintentional engagement of unguided thoughts^9,18,19^. This distinction has proved useful with individuals reporting more spontaneous mind wandering on the difficult (standard) version of the SART and more deliberate mind wandering on the easy (sequential) version of the SART^20^, supporting the notion of an act of control over deliberate mind wandering.

Recently, research has revealed a link between ADHD symptoms or traits and frequent experiences of mind wandering^21–23^. Attention Deficit Hyperactivity Disorder^24^ is a widely-diagnosed childhood-onset neurodevelopmental disorder with prevalence rates of 5–10% in childhood^25^ and 4.4% in adulthood^26^. ADHD manifests itself in three presentations: Predominantly Inattentive (ADHD-I), Predominantly Hyperactive/Impulsive (ADHD-H) and combined (ADHD-C)^24^. Although clinical diagnosis of ADHD involves determining either the absence or presence of diagnosis based on the number of symptoms present, individuals could be affected by the impairments associated with ADHD at sub-clinical levels^27,28^. Indeed, characterising inattention, hyperactivity and impulsivity as continua has been supported by taxometric studies^29,30^.

Whilst previous research has revealed a link between ADHD symptoms or traits and frequent experiences of mind wandering they have not considered the relationship between mind wandering and the core symptoms of inattention, hyperactivity and impulsivity individually. This might be because the DSM-V lists mind wandering as being related to inattention only. The following items appear only in the inattention symptom list: “Often does not seem to listen when spoken to directly (*e*.*g*., mind seems elsewhere, even in the absence of any obvious distraction”^24^ and “Is often easily distracted by extraneous stimuli (for older adolescents and adults, may include unrelated thoughts.” This is also consistent with the finding that, whilst inattention, hyperactivity and impulsivity have been associated with poor executive functioning^31^, only inattention is theoretically and empirically related to impairments in working memory^32–37^, the executive function linked to mind wandering. However, contrary to the notion that mind wandering is closely associated with inattention, Shaw and Giambra^22^ reported that experiences of spontaneous mind wandering were more frequent in individuals with higher self-reported hyperactive (*e*.*g*. fidgeting)^38^ and impulsive^39^ traits (they did not investigate inattentive traits). Indeed, Seli *et al*.^23^ have proposed that the distinction between spontaneous and deliberate mind wandering could be crucial in terms of understanding the aspects of ADHD symptomatology. For example, it is possible that hyperactive and impulsive traits are more related to spontaneous mind wandering, whilst inattention is more associated with deliberate mind wandering. This relationship might also be dependent on task difficulty and whether the cognitive underload or overload view of the SART is correct. For example, under the cognitive underload view of the SART^15,40–42^, the task effectively promotes inattention. Thus given the nature of the task and evident issues those with inattention have with working memory, cognitive effort^43^ and sustained attention^38,44^, it is reasonable to think that high inattentive traits would be associated with frequent deliberate mind wandering, especially in the easy condition. Alternatively, under the cognitive overload view, performing the SART requires constant monitoring over time, using executive resources, and as such inattentive individuals would be expected to report frequent spontaneous mind wandering, especially when the task is more difficult (when the digits are presented randomly) due to their limited working memory capacity^35,36^. Thus, the present study could provide results that are informative to the mind wandering literature. In terms of understanding mind wandering itself and not just the SART, findings showing that ADHD-like traits are associated with spontaneous mind wandering would be inconsistent with the notion that spontaneous mind wandering is resource demanding since ADHD and its traits are associated with poorer executive functions and thus have access to fewer resources^31^.

The aim of the present work was to investigate the relationship between self-reported traits of inattention, hyperactivity and impulsivity and types of mind wandering in a non-clinical sample of high functioning adults. Participants completed an ADHD questionnaire reporting their behavioural tendencies related to inattentive, hyperactive and impulsive traits. In addition to the ADHD questionnaire, we also measured experiences of spontaneous mind wandering and tendency to engage in deliberate mind wandering during both difficult and easy versions of the Sustained Attention to Response Task.

## Results

### Sample

**Figure 1:**
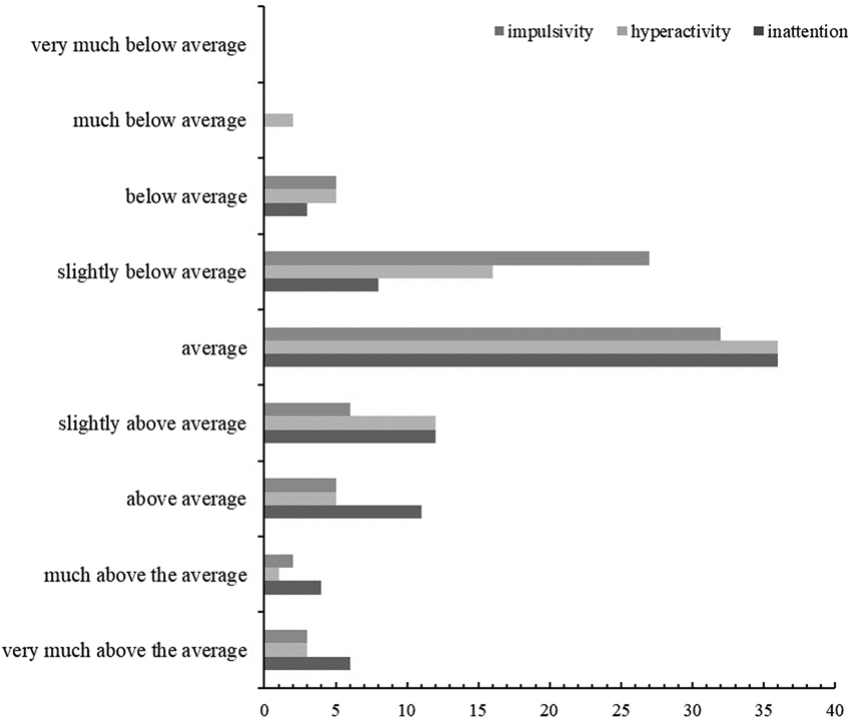
Number of participants falling into each category based on CAARS guidelines.

Scores from the CAARS revealed that 25% of participants scored above average on the ADHD index (*M* = 53.15, *SD* = 9.75). For individual symptoms, the percentage of participants scoring above the average was 53% for inattentive (*M* = 54.41, *SD* = 9.22), 6% for hyperactive (*M* = 50.06, *SD* = 8.68) and 60% for impulsive symptoms (*M* = 49.12, *SD* = 9.30). Five participants for inattentive, three participants for hyperactive and three participants for impulsive traits scored above the t-score of 70 which is the cutoff point indicating clinically significant problems (see Fig. 1 for more detail). One participant also reported a previous ADHD diagnosis whereas two participants preferred not to state. Please see Table 1 in the supplementary material for detailed participant characteristics. Please note that the data from one participant was missing due to an incomplete data set (the experimental program was shut down half way through the easy SART due to a technical error on the computer and the data were not recorded as the task had not been completed). One participant’s scores for deliberate and spontaneous mind wandering was removed due to unreliable task performance (the participant reported probe questions but did not respond to digits 1–9 except for 3 for more than half the trials). Total data loss was 1.25%.

**Table 1:** Correlations between variables.

	N	Mean	SD	1	2	3	4	5	6	7	8	9
1. Inattention	80	54.41	9.22	—								
2. Hyperactivity	80	50.06	8.68	0.51^**^	—							
3. Impulsivity	80	49.13	9.30	0.45^**^	0.56^**^	—						
4. Spontaneous MW (difficult condition)	79	6.70	4.16	0.38^**^	0.34^**^	0.05	—					
5. Spontaneous MW (easy condition)	78	6.71	4.31	0.19	0.27^*^	−0.06	0.49^**^	—				
6. Deliberate MW (difficult condition)	79	3.61	3.38	−0.02	0.05	0.16	−0.40^**^	−0.19	—			
7. Deliberate MW (easy condition)	78	5.27	4.52	0.03	0.16	0.25^*^	0.03	−0.30^**^	0.43^**^	—		
8. On**-**task (difficult condition)	80	9.60	4.25	−0.37^**^	−0.35^**^	−0.19	−0.62^**^	−0.34^**^	−0.41^**^	−0.39^**^	—	
9. On**-**task (easy condition)	79	7.96	5.24	−0.19	−0.35^**^	−0.17	−0.43^**^	−0.57^**^	−0.21	−0.62^**^	0.62^**^	—

^*^p < 0.05, ^**^p < 0.01.

### Task Difficulty and Mind Wandering

We conducted a 2 (condition: easy, difficult) × 3 (Mind Wandering Type: spontaneous, deliberate and on-task) repeated-measures ANOVA to evaluate the effect of task difficulty on mind wandering. A Mind Wandering Type main effect was found, *F*(2, 77) = 17.52 *p* < 0.001, *η*^2^ = 0.19. Bonferroni corrected pairwise comparisons revealed that participants reported more spontaneous than deliberate mind wandering (*Mdiff* = 2.29, *SE* = 0.64, *p* = 0.002, *d* = 0.66), an*d*, more on-task reports than spontaneous mind wandering (*Mdiff* = 2.06, *SE* = 0.80, *p* = 0.037, *d* = 0.53). There were also more on-task reports than deliberate mind wandering reports (*Mdiff* = 4.35, *SE* = 0.76, *p* < 0.001, *d* = 1.16).

**Figure 2:**
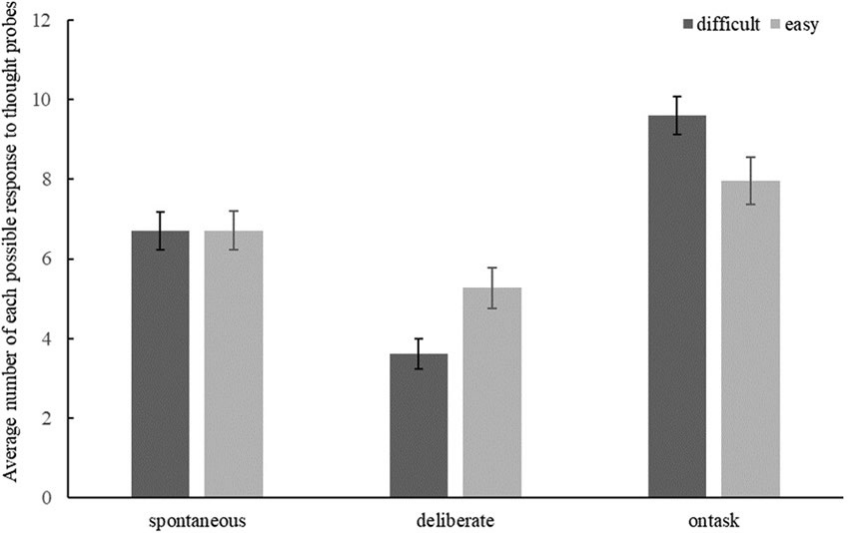
Average number of participants’ reports of spontaneous and deliberate mind wandering and on task events by condition. Error bars indicating the standard error.

Additionally, a Condition x Mind Wandering Type interaction was found, *F* (2, 77) = 7.23, *p* = 0.001, *η*^2^ = 0.09. Bonferroni corrected paired samples t-tests were conducted to evaluate the Condition X Mind Wandering Type interaction (Fig. 2). Participants reported more deliberate mind wandering in the easy condition (*M* = 5.27, *SE* = 0.51 vs. *M* = 3.64, *SE* = 0.38, *t* (77) = 3.33, *p* = 0.001, *d* = 0.41). However, levels of spontaneous mind wandering between the easy and difficult conditions did not differ, *t* (77) = 0.16, *p* = 0.87. There were also more on-task reports in the difficult than in the easy condition (*M* = 9.48, *SE* = 0.47 vs. *M* = 7.96, *SE* = 0.59, *t* (78) = −3.19, *p* = 0.002, *d* = 0.32).

Furthermore, in the difficult condition, participants reported more spontaneous than deliberate mind wandering (*M* = 6.70, *SE* = 0.47 vs. *M* = 3.61, *SE* = 0.38, *t* (78) = 4.35, *p* < 0.001, *d* = 0.82) whereas in the easy condition there was no significant difference between spontaneous and deliberate mind wandering, *t* (77) = −1.78, *p* = 0.078. On task reports were higher than both spontaneous (*M* = 9.70, *SE* = 0.47 vs. *M* = 6.70, *SE* = 0.47, *t* (78) = −3.49, *p* = 0.001, *d* = 0.72) and deliberate (*M* = 3.61, *SE* = 0.38, *t* (78) = 8.48, *p* < 0.001, *d* = 1.61) mind wandering in the difficult condition whereas on-task reports did not significantly differ from spontaneous *(t* (77) = −1.38, *p* = 0.17), and, deliberate (*t* (77) = 2.77, *p* = 0.007; non-significant following Bonferroni correction) mind wandering in the easy condition. In order to investigate the overall amount of mind wandering (the sum of spontaneous and deliberate) in the easy and difficult conditions, we also ran a pairwise comparison. There was more mind wandering reports in the easy than the difficult condition (*M* = 11.97, *SE* = 0.59 vs. *M* = 4.08, *SE* = 0.46, *t* (77) = −3.22, *p* = 0.002, *d* = 1.70). That is, the task difficulty manipulation modified type of mind wandering by reducing deliberate mind wandering in the difficult condition. In summary, overall mind wandering was higher in the easy compared to the difficult condition, and, participants reported more on-task reports in the difficult condition. We also observed more deliberate mind wandering in the easy condition. Please note that the accuracy and reaction time data for the SART were not analysed.

### ADHD Symptoms and Mind Wandering

We first examined the bivariate correlations between the independent and dependent variables (Table 1). We then conducted multiple regression analyses with the enter method for each step to determine how CAARS scores for each individual trait (inattention, hyperactivity and impulsivity) were related to the different types of mind wandering in the easy and difficult task conditions. Variables were included in the same order for all models. The variable order was as follows: inattention, hyperactivity and impulsivity. We first entered inattention scores into the model since it seems to be the most likely candidate to predict types of mind wandering, as noted above. We then entered the scores for hyperactivity^38^ and impulsivity^39^ related traits. We also aimed to see whether the scores for inattentive traits alone would predict types of mind wandering and if so, would the predictive value of inattention be shared when controlling for other traits (hyperactivity and impulsivity). We used Bayes Factors (B) following the procedures of Dienes^45^ with the proposed cut-offs^46^ to assess the strength of evidence in support of hypotheses when the p value was not significant. In all cases where a Bayes Factor is given we modelled the predictions of the theory of some evidence for a relationship with a half-normal whose mean and standard deviation values were taken from a significant predictor of relevant regression analysis.

#### Spontaneous mind wandering

We conducted hierarchical regression analysis to test if the self-reported traits of ADHD measured by CAARS predicted types of mind wandering in both difficult and easy task conditions. For the difficult condition (Table 2), at stage one, scores for inattentive traits significantly predicted spontaneous mind wandering, *F*(1, 78) = 13.14, *p* = 0.001. At stage two, inattentive scores remained a significant predictor and the model explained 15% of the variation, *F*(2, 78) = 7.93, *p* = 0.001. At stage three, with the addition of impulsivity scores the model explained more of the variation (23%), *F*(3, 78) = 7.30, *p* < 0.001. Interestingly the relationship between spontaneous mind wandering and impulsivity traits was negative where the greater the reports of impulsivity, the fewer instances of spontaneous mind wandering reported.

**Table 2:** Summary of the regression model for inattention, hyperactivity and impulsivity scores of CAARS on spontaneous mind wandering in the difficult condition.

	Variable	*b*	SE*b*	β	*t*	*R* ^*2*^	*R*^*2*^ change	Semi-partial correlation
Step 1						0.15	0.15**	
	inattention	0.17	0.05	0.38	3.63**			
Step 2						0.17	0.03	
	inattention	0.13	0.06	0.28	3.33*			0.26
	hyperactivity	0.09	0.06	0.19	1.57			
Step 3						0.23	0.05*	
	inattention	0.16	0.055	0.34	2.33**			0.31
	hyperactivity	0.16	0.06	0.33	2.46*			0.27
	impulsivity	−0.13	0.06	−0.29	−2.28*			−0.25

^*^p < 0.05, ^**^p < 0.01.

For the easy condition (Table 3), at stage one the model was not significant and inattention scores of CAARS alone did not predict spontaneous mind wandering. With the addition of the scores for hyperactive traits of CAARS, the model became significant and explained 8% of the variation, *F*(2, 77) = 3.13, *p* = 0.049. When scores for impulsivity related traits were added, hyperactivity scores became significant while inattention scores were still non-significant (*p* = 0.276, *B*_H(0, 0.07)_ = 0.06; the population prior was calculated from the variable hyperactivity in the same model). As with spontaneous mind wandering in the difficult condition, CAARS scores for impulsivity traits had a negative relationship with spontaneous mind wandering. The model explained more of the variation, *F*(3, 77) = 4.53, *p* = 0.006. Interestingly, this shows that whilst spontaneous mind wandering was predicted by the reports of inattentive traits in the difficult condition, it was not in the easy condition whereas both the scores for impulsivity and hyperactive traits predicted spontaneous mind wandering in both the difficult and easy conditions.

**Table 3:** Summary of the regression model for inattention, hyperactivity and impulsivity scores of CAARS on spontaneous mind wandering in the easy condition.

	Variable	*b*	SE*b*	β	*t*	*R* ^2^	*R*^2^ change	Semi-partial correlation
Step 1						0.04	0.04	
	inattention	0.09	0.05	0.19	1.68			
Step 2						0.08	0.04	
	inattention	0.03	0.06	0.07	0.52			0.06
	hyperactivity	0.12	0.07	0.24	1.82			0.21
Step 3						0.16	0.08*	
	inattention	0.07	0.06	0.14	1.10			0.13
	hyperactivity	0.20	0.07	0.40	2.85**			0.31
	impulsivity	−0.16	0.06	−0.35	−2.62*			−0.29

^*^p < 0.05, ^**^p < 0.01.

#### Deliberate mind wandering

None of the models were significant at any stage: (1) Difficult condition: [Model 1: *F*(1, 77) = 0.03, *p* = 0.861; Model 2: *F*(2, 78) = 0.21, *p* = 0.810; Model 3: *F*(3, 78) = 0.92, *p* = 0.434] and; (2) easy condition [Model 1: *F*(1, 77) = 0.06, *p* = 0.906; Model 2: *F*(2, 77) = 1.12, *p* = 0.331; Model 3: *F*(3, 77) = 1.99, *p* = 0.123].

#### Reports of on-task

We conducted hierarchical regression analysis to test if the self-reported traits of ADHD measured by CAARS predicted the number of times participants reported being on-task for both the difficult and easy task conditions. The population prior for the Bayes factor calculation was taken from the variable inattention in the relevant model. For the difficult condition (Table 4), at stage one, scores for inattentive traits significantly predicted the number of on-task reports, *F*(1, 79) = 12.37, *p* = 0.001. At stage two, the model was significant (*F*(2, 79) = 8.04, *p* = 0.001) where inattentive traits remained significant when hyperactive traits were a non-significant but insensitive predictor (*p* = 0.072, *B*_H(0, 0.06)_ = 0.60). At stage three, with the addition of impulsive traits, the model explained 18% of the variation, *F*(3, 79) = 5.44, *p* = 0.002. Inattentive traits were the only significant predictor, hyperactive traits resulted in an insensitive result (*p* = 0.06 *B*_H(0, 0.06)_ = 0.73) and there was strong evidence for no relationship for the impulsive traits (*p* = 0.539, *B*_H(0, 0.06)_ = 0.14).

**Table 4:** Summary of the regression model for inattentive, hyperactive and impulsive traits of CAARS on the number of on-task reports where mind wandering is absent in the difficult condition.

	Variable	*b*	SE*b*	β	*t*	*R* ^2^	*R*^2^ change	Semi-partial correlation
Step 1						0.14	0.14**	
	inattention	−0.17	0.05	−0.37	−3.52**			−0.37
Step 2						0.17	0.04	
	inattention	−0.12	0.06	−0.26	−2.16*			−0.24
	hyperactivity	−0.11	0.06	−0.22	−1.83			−0.20
Step 3						0.18	0.004	
	inattention	−0.13	0.06	−0.28	−2.24*			−0.25
	hyperactivity	−0.13	0.07	−0.26	−1.91			−0.21
	impulsivity	0.04	0.06	0.08	0.62			0.07

^*^p < 0.05, ^**^p < 0.01.

For the easy condition (Table 5), at stage one, for the model where only inattentive traits were entered, the number of on-task reports was not significant, *F*(1, 78) = 2.81, *p* = 0.10. At stage two (*F*(2, 78) = 5.23, *p* = 0.007), hyperactive traits were a significant predictor but there was strong evidence that inattentive traits were non-significantly related to on-task reports (*p* = 0.903, *B*_H(0, 0.07)_ = 0.09). At stage three, the model explained 12% of the variation, *F*(3, 78) = 3.47, *p* = 0.02 with the addition of impulsive traits. Hyperactive traits were again the only significant predictor whereas there was strong evidence for no relationship with on task reports and inattentive (*p* = 0.855, *B*_H(0, 0.07)_ = 0.09) and impulsive (*p* = 0.781, *B*_H(0, 0.07)_ = 0.10) traits.

**Table 5:** Summary of the regression model for self-reported inattentive, hyperactive and impulsive traits of CAARS on the number of on-task reports where mind wandering is absent in the easy condition.

	Variable	*b*	SE*b*	β	*t*	*R* ^2^	*R*^2^ change	Semi-partial correlation
Step 1						0.04	0.04	
	inattention	−0.11	0.06	−0.19	−1.68			
Step 2						0.12	0.09^**^	
	inattention	−0.01	0.07	−0.02	−0.12			−0.01
	hyperactivity	−0.21	0.08	−0.34	−2.72^**^			−0.30
Step 3						0.12	0.001	
	inattention	−0.01	0.07	−0.02	−0.18			−0.02
	hyperactivity	−0.22	0.09	−0.36	−2.57^*^			−0.28
	impulsivity	0.02	0.08	0.04	0.28			0.03

^*^p < 0.05, ^**^p < 0.01.

## Discussion

The aim of the present experiment was to investigate the relationship between spontaneous and deliberate mind wandering and self-reported traits of inattention, hyperactivity and impulsivity. A sample of undiagnosed adults (mainly university students) was used to explore this relationship given the evidence showing that both ADHD and the individual core symptoms are best described as being on a continuum^27,28^. Mind wandering was measured using the probe-caught method during performance of the Sustained Attention to Response Task (SART). Moreover, we measured mind wandering during both standard (difficult) and less challenging (sequential) versions of the SART. The results revealed that inattentive traits were related to spontaneous mind wandering but only under difficult conditions; when the task was easy there was strong evidence for no relationship between inattention and spontaneous mind wandering. Hyperactive traits were related to spontaneous mind wandering in both the easy and the difficult conditions but in both cases only when impulsive traits were added to the model. Impulsive traits also predicted spontaneous mind wandering in both the easy and the difficult conditions. Finally, none of the ADHD related traits were related to deliberate mind wandering in the difficult or easy conditions, which is consistent with the findings of Seli *et al*.^23^. Importantly, the finding that spontaneous mind wandering was predicted by inattentive traits in the difficult condition only but was predicted by hyperactivity/impulsivity in both conditions is inconsistent with mind wandering being represented only in the inattention symptom list of the DSM-V^24^.

The strong evidence for no relationship between self-reported inattentive traits and mind wandering under easy task conditions is a surprising result given that hyperactive/ impulsive traits uniquely predicted spontaneous mind wandering in the same condition, and given that the DSM-V refers to mind wandering related symptoms only under ADHD-I^24^. This finding suggests that spontaneous mind wandering in the easy task condition might be more related to failures in the executive function of inhibition. In contrast, the finding that for individuals reporting high levels of inattentive traits, spontaneous mind wandering is present when the task is difficult, suggests that spontaneous task unrelated thoughts may be triggered by the need for extra processing and effort on a task. Given the stronger relationship between self-reported inattentive traits and working memory than between hyperactive/impulsive traits and working memory even in non-clinical samples^36^, it is possible that spontaneous mind wandering in the difficult condition may be related to working memory limitations. That is, when resources are consumed by an ongoing task, working memory may be less able to prevent intrusive unrelated thoughts. Thus, our results point to potentially different underlying mechanisms for the same type of mind wandering in different conditions. Further studies with clinical samples would be beneficial to establish if this finding holds at clinical levels of ADHD.

Impulsive traits had a negative relationship with spontaneous mind wandering in both the easy and difficult conditions, indicating that the higher the impulsivity scores, the less mind wandering participants experienced. This was an unexpected result. Before going on to present possible explanations for this, we need to address a potential methodological explanation. The probe caught method itself could have influenced the results: Individuals reporting high impulsivity traits may have been more likely to choose the first possible probe option on the screen (on task was the option presented first). However, in our data, although impulsive traits were negatively related to mind wandering, there was strong evidence for no relationship between reported impulsivity traits and reports of being on-task in both the easy and difficult task conditions, rendering invalid this explanation of the data. Nevertheless, in future experiments it might be preferable to measure mind wandering by presenting the possible probe response options (e.g. deliberate, spontaneous, on task) in random order.

Aside from the practical methodological design explanation, it is possible that the negative relationship between impulsivity and spontaneous mind wandering might have something to do with the SART itself. It has been argued that the SART is a measure of the ability to inhibit impulsive responses^13^. Since research has revealed a decrease in mind wandering during challenging tasks^47^ it is possible that if those with impulsive traits find the task more difficult (precisely because the task is a measure of impulse control), they would be expected to report fewer incidences of mind wandering and thus a negative relationship between impulsivity and mind wandering would be observed.

An alternative explanation for this negative relationship is that individuals reporting high impulsive traits may be less able to catch spontaneous mind wandering reliably. Consistently, self-reported impulsive traits have been shown to be positively correlated with error awareness^48^, and, impulsive errors (measured by the ability to inhibit impulsive responding during SART) increase when participants are not aware of mind wandering^2^. A methodological issue in mind wandering research is that the studies rely on participants’ self-reports, however, mind wandering may continue for some time before it reaches awareness^1,49,50^. Therefore, a possible impairment with meta-awareness of spontaneous mind wandering in those reporting high levels of impulsivity traits may result in under recognized thus unreported mind wandering events. Further research is needed to investigate the role of meta-awareness, impulsivity and spontaneous mind wandering.

### On the control of spontaneous and deliberate mind wandering

The literature suggests that mind wandering competes with limited cognitive resources^1,2^. Therefore, it would be expected that individuals would experience more mind wandering when the task requires fewer cognitive resources (easy task) as there would be more available cognitive resources for mind wandering (attentional resources account)^1,51,52^. Consistent with the attentional resources account, our findings suggest that the overall rate of mind wandering was higher in the easy compared to the difficult condition. However, this was driven by a decrease in deliberate mind wandering in the difficult condition (and an increase in on-task reports), whereas spontaneous mind wandering did not differ between conditions. Such a pattern could be interpreted as showing that only deliberate mind wandering competes with the demands of primary task for cognitive resources. This indicates that participants are more likely to control their deliberate mind wandering when they are aware of task difficulty, but also that it might not be possible to exert control over spontaneous mind wandering. Our results are consistent with Seli, Risko, Smilek, & Schacter^17^ who suggested that deliberate and spontaneous mind wandering reflect different attentional control networks. However, Seli *et al*. reported that individuals had more spontaneous mind wandering in the difficult compared to the easy SART condition, a finding not replicated in the present study. A possible account for the different findings is that Seli *et al*. compared mind wandering levels in easy and difficult conditions using a between-subjects design whereas a repeated-measures design was used for the present study to minimize the effect of individual differences in the levels of mind wandering experienced. Indeed in our study task order analysis showed that participants reported more spontaneous mind wandering in the easy condition when the difficult condition was run first (*Mdiff* = 2.5, *p* = 0.007) while there was no effect of task order for the difficult condition, suggesting some level of support for Seli *et al*.’s finding.

In conclusion, we measured the relationship between the frequency and type of mind wandering and self-reported inattentive, hyperactive and impulsive traits of ADHD at subclinical levels. We found that all ADHD related traits (inattention, hyperactivity and impulsivity) predicted spontaneous but not deliberate mind wandering, and that spontaneous mind wandering is not uniquely associated with inattentive traits. Moreover, inattentive and hyperactive traits were shown to be differentially linked to spontaneous mind wandering: spontaneous mind wandering was predicted by hyperactive traits in both easy (sequential) and difficult (standard) SART while inattentive traits predicted spontaneous mind wandering only when the task was cognitively challenging (standard SART, difficult condition), which suggests that spontaneous mind wandering might have different underlying causes, depending on task difficulty.

## Method

### Participants

Data were collected from an opportunity sample of 80 undiagnosed individuals. Participants were mainly students from Bournemouth University (39 undergraduate and 39 postgraduate and 3 other sources of employment). Participants were recruited through Bournemouth University’s research participation system or an advertisement placed around the university listing the inclusion criteria of normal or corrected vision and an age limit (between 18 and 40). We included Bayes factors so that there would be an assessment of the sensitivity of the data to distinguish H0 and H1. Sample size was determined by the availability of participants. Recruited participants were aged between 18 to 37 (*M* = 24.46, *SD* = 0.50) with self-reported normal or corrected vision. There were 30 male (*M* = 26.10, *SD* = 4.42) and 50 female (*M* = 23.56, *SD* = 4.23) participants. Participants received £10 for their involvement. All experimental protocols were approved by Bournemouth University ethics committee. We confirm that all participants were provided with a consent form. We also confirm that all methods were performed in accordance with the relevant guidelines and regulations. Data collection and analyses were carried out in accordance with the approved study protocol and the guidelines of The Helsinki Declaration and Code of Human Research Ethics and The British Psychological Society.

### Materials

#### Connors’ Adult ADHD Rating Scale: Short Version (CAARS-S:S)

ADHD traits were assessed using CAARS-S:S^53^. Raw scores for inattention, hyperactivity and impulsivity symptoms are transformed into t-scores to make a comparison across participants. T scores range between 28 (lowest) to 90 (highest) calculated based on the age and gender.

#### Sustained Attention to Response Task (SART)

The Sustained Attention to Response Task was used to induce mind wandering. Two versions of the SART (standard and easy) were used to manipulate task difficulty as variation in task demands are reported to affect the measured frequency of deliberate and spontaneous mind wandering^9^.

- The Standard Sustained Attention to Response Task (SART; Difficult):
  On each trial, a single digit (1–9) was centrally presented for 250 ms. Then, an “x” mask was presented for 900 ms (total trial duration = 1150 ms). On each block, a digit was randomly chosen from 1 to 9 without replacement, and was presented in black on a white background. Therefore, each digit was presented equally across the experimental trials. Participants were required to make a button press for every digit except 3. The presentation of the digit 3 required withholding the button press response. Participants were instructed to be as fast and as accurate as possible. The digits were presented in Courier New font. The digit sizes varied across all trials to control the familiarity effect. There were five possible font sizes (120, 100, 94, 72, and 48 points). Every nine trials four of the possible font sizes appeared twice and one appeared once (determined randomly).
  A thought probe was randomly presented within every 45 trials (five blocks of 9 digits), asking “Which of the following responses best characterises your mental state RIGHT NOW?” Participants were expected to choose one of the following possible responses (1) On task (2) Intentionally mind wandering (3) Unintentionally mind wandering. Experimental trials started with nine dummy trials with no thought probe presented to ensure that the thought probe was presented after several trials. Participants performed a total of 900 experimental trials (and nine dummy trials) with 20 thought probes following 18 practice trials with two thought probes.
- The Sequential Sustained Attention to Response Task (SART; Easy):

The procedure of the easy task was identical to the standard version of SART except that the digit presentation was in a sequential order (1 through 9), allowing it to be predictable. The task was adapted from Seli *et al*.^9^.

### Procedure

Initially, informed consent was obtained from all participants. After giving informed consent, participants were asked to sit in front of a computer at a distance of 50 cm. The presentation order of the SART and CAARS was pseudo randomised between participants. The SART conditions (easy and difficult) were also counterbalanced across participants.

Prior to completing the SART, participants received detailed instructions on the thought probes. Participants were informed that being on task means that either they were not thinking anything, or they were thinking about things related to the task (e.g. thoughts about their performance on the task, thoughts about the digits, or thoughts about their response), whereas mind wandering means that they were thinking about something completely unrelated to the task (e.g. thoughts about what to eat for dinner, thoughts about an upcoming test or friends). It was also made clear that participants were required to focus on the task in order to achieve the task performance. They were then informed that if they experience any mind wandering, they should indicate whether the mind wandering occurred intentionally (deliberately) or unintentionally (spontaneously). It was explained that mind wandering may occur unintentionally but may continue intentionally or unintentionally. Providing that participants are motivated to perform the task, mind wandering could/should initiate only in a spontaneous, unintentional manner.

### Data availability

The datasets generated during and/or analysed during the current study are available from the corresponding author on reasonable request.

## Electronic supplementary material

Supplementary Information
