# Supplementary material for: Probe-caught spontaneous and deliberate mind wandering in relation to self-reported inattentive, hyperactive and impulsive traits in adults
Source: Sci Rep. 2018 Mar 7;8:4113. doi: 10.1038/s41598-018-22390-x (PMC5841345; doi:10.1038/s41598-018-22390-x)
Supplement: Supplementary file 1 — Supplementary Information [file 41598_2018_22390_MOESM1_ESM.pdf]

Probe-caught spontaneous and deliberate mind wandering in relation to self-reported inattentive, hyperactive and impulsive traits in adults

Gizem Arabacı* and Benjamin A. Parris

Department of Psychology

Bournemouth University

Address for correspondence:

Gizem Arabacı

Department of Psychology,

Bournemouth University

Talbot Campus

Fern Barrow

Poole

Dorset UK

BH12 5BB

01202 524111

garabaci@bournemouth.ac.uk

Supplementary Material

Table 1

Demographic information, CAARS t-scores and mind wandering scores for each participant

					CAARS scores				Standard SART probe responses			Sequential SART probe response		
Participant number	Age	Occupation	Gender	Previous diagnosis	Inattention	Hyperactivity	Impulsivity	Index	OnTask	Deliberate	Spontaneous	OnTask	Deliberate	Spontaneous
1	22	postgraduate	female	no	51	49	55	53	9	11	0	11	3	6
2	21	undergraduate	male	no	61	47	51	60	12	8	0	7	5	8
3	19	undergraduate	male	yes	74	59	51	70	9	1	10	6	4	10
4	19	undergraduate	male	no	64	56	42	53	13	1	6	17	1	2
5	20	undergraduate	female	no	63	57	46	73	19	1	0	12	1	7
6	22	undergraduate	male	no	70	59	58	68	4	8	8	8	5	7
7	26	postgraduate	female	no	45	40	43	47	18	1	1	16	3	1
8	27	postgraduate	female	no	63	54	61	61	9	3	8	18	0	2
9	21	undergraduate	male	no	57	59	48	53	3	3	14	0	2	18
10	24	postgraduate	male	no	51	34	38	45	11	4	5	7	8	5
11	36	postgraduate	male	no	59	47	52	48	10	1	9	8	3	9
12	20	undergraduate	male	no	57	51	79	61	14	3	3	9	7	4
13	24	postgraduate	female	no	47	53	48	45	6	3	11	4	2	14
14	21	undergraduate	female	no	75	63	61	71	10	1	9	10	4	6
15	18	undergraduate	female	no	60	72	61	65	5	5	10	0	7	13
16	20	undergraduate	male	no	80	74	77	90	5	10	5	3	8	9
17	20	undergraduate	female	no	48	43	46	48	10	3	7	10	2	8
18	20	undergraduate	male	no	54	47	42	43	8	6	6	2	13	5
19	18	undergraduate	male	no	47	44	40	36	2	17	1	3	17	0

20	28	postgraduate	male	no	51	43	43	57	17	2	1	19	1	0
21	26	postgraduate	female	no	51	37	58	42	13	5	2	20	0	0
22	29	postgraduate	female	no	37	43	46	40	13	2	5	9	4	7
23	29	postgraduate	female	no	69	63	61	61	0	0	20	0	20	0
24	24	postgraduate	male	no	47	47	42	47	11	4	5	8	5	7
25	26	postgraduate	female	no	48	40	43	53	18	2	0	10	2	8
26	26	postgraduate	male	no	44	38	42	43	9	11	0	18	2	0
27	19	undergraduate	male	no	51	50	48	57	7	6	7	5	7	8
28	18	undergraduate	male	no	54	56	45	50	6	4	10	2	11	7
29	18	undergraduate	male	no	67	68	74	70	7	11	2	1	16	3
30	20	undergraduate	male	no	61	38	45	60	8	4	8	8	7	5
31*	20	undergraduate	male	no	61	44	55	55	.	.	.	.	.	.
32	21	undergraduate	female	no	57	49	52	53	14	1	5	6	7	7
33	34	postgraduate	female	no	55	56	42	59	11	3	6	11	3	6
34	28	postgraduate	male	no	74	56	42	58	5	0	15	9	5	6
35	23	postgraduate	male	no	54	51	40	47	5	2	13	1	0	19
36	26	postgraduate	female	no	54	49	38	45	9	5	6	4	7	9
37	28	postgraduate	male	no	67	50	58	58	11	0	9	8	6	6
38	26	postgraduate	female	no	51	49	43	44	10	1	9	5	9	6
39	23	postgraduate	male	no	51	44	40	43	9	7	4	5	9	6
40**	19	undergraduate	female	no	51	34	40	40	19	1	0	.	.	.
41	26	undergraduate	female	no	61	50	58	60	7	4	9	10	5	5
42	22	undergraduate	female	no	64	62	51	62	7	5	8	13	2	5
43	25	undergraduate	male	no	41	59	40	47	7	2	11	9	4	7
44	29	undergraduate	female	no	45	60	55	48	13	2	5	4	6	10
45	24	undergraduate	male	no	54	50	48	60	7	8	5	5	7	8
46	35	undergraduate	female	no	45	41	55	48	11	0	9	16	2	2
47	28	postgraduate	male	no	47	53	55	52	11	5	4	7	2	11
48	27	postgraduate	female	no	48	46	43	47	9	1	10	4	3	13
49	24	postgraduate	male	no	51	50	40	38	9	5	6	6	8	6
50	28	postgraduate	male	no	44	47	48	53	12	6	2	14	6	0
51	29	postgraduate	male	no	64	38	42	48	6	2	12	5	0	15

52	21	undergraduate	male	no	54	56	45	53	15	0	5	14	0	6
53	21	undergraduate	male	no	41	38	37	36	11	1	8	6	8	6
54	27	postgraduate	male	no	57	50	55	58	5	5	10	4	6	10
55	21	undergraduate	male	no	39	53	64	57	14	1	5	0	18	2
56	26	undergraduate	male	no	44	41	42	48	11	0	9	14	0	6
57	23	undergraduate	male	no	44	50	48	43	16	1	3	12	0	8
58	25	postgraduate	male	no	39	43	38	39	16	2	2	18	0	2
59	35	other	female	no	47	65	48	55	10	1	9	6	8	6
60	29	undergraduate	male	no	61	47	42	60	6	5	9	12	5	3
61	25	postgraduate	female	no	54	49	43	48	9	0	11	7	2	11
62	21	undergraduate	male	no	61	59	55	62	11	1	8	8	1	11
63	22	undergraduate	male	not stated	74	74	67	77	4	0	16	1	0	19
64	25	other	male	no	47	53	42	52	8	6	6	9	3	8
65	19	undergraduate	male	no	44	41	40	42	16	2	2	15	3	2
66	21	undergraduate	male	no	54	47	48	58	10	2	8	10	2	8
67	27	postgraduate	female	no	57	43	46	53	2	3	15	8	3	9
68	32	postgraduate	female	no	49	62	68	56	2	12	6	0	16	4
69	27	postgraduate	female	no	51	46	46	50	5	10	5	5	12	3
70	26	postgraduate	female	no	57	43	38	45	8	2	10	3	4	13
71	21	undergraduate	male	no	47	47	42	45	11	4	5	8	2	10
72	29	postgraduate	male	no	51	50	42	53	12	0	8	8	4	8
73	27	postgraduate	female	no	57	46	52	57	4	3	13	2	9	9
74	25	postgraduate	male	no	57	47	48	52	11	1	8	6	9	5
75	37	postgraduate	male	no	56	54	58	63	14	1	5	19	0	1
76	22	postgraduate	male	no	66	43	58	58	12	4	4	7	9	4
77	22	undergraduate	male	not stated	57	49	43	57	14	3	3	13	4	3
78	27	postgraduate	female	no	48	49	49	45	9	8	3	1	11	8
79	27	postgraduate	male	no	44	44	55	53	14	2	4	14	5	1
80	21	undergraduate	male	no	51	47	40	42	8	4	8	3	6	11

* Mind wandering scores for participant 31 were removed.
** Mind wandering scores for participant 40 were not recorded due to technical difficulties.
